# Metformin in gynecological disorders: pathogenic insights and therapeutic implications

**DOI:** 10.3389/fphar.2025.1526709

**Published:** 2025-04-22

**Authors:** Ping Nie, Minghua Wang, Yan Mo, Hong Zhou, Qingbing Zha, Gendie E. Lash, Ping Li

**Affiliations:** ^1^ Department of Pathology, Jinan University School of Medicine, Guangzhou, China; ^2^ Department of Pathology, Longgang District People’s Hospital, Shenzhen, China; ^3^ Center of Reproductive Medicine, Jinan University First Affiliated Hospital, Guangzhou, China; ^4^ Guangdong Provincial Key Laboratory of Spine and Spinal Cord Reconstruction, Jinan University Fifth Affiliated Hospital (Heyuan Shenhe People’s Hospital), Heyuan, China; ^5^ Guangzhou Institute of Pediatrics, Guangzhou Women and Children’s Medical Center, Guangzhou Medical University, Guangzhou, China

**Keywords:** metformin, polycystic ovary syndrome, endometriosis, premature ovarian failure, gynecologic malignancies, ovary, uterus

## Abstract

Metformin, the most widely used anti-diabetic drug, has been demonstrated to exert various effects, including antioxidant, anti-inflammatory, anti-tumor, and cardioprotective properties. Due to its affordability and low toxicity profile, metformin is increasingly used to prevent or treat a wide range of gynecological disorders, as evidenced by epidemiological studies, clinical trials, and animal and *in vitro* studies. Trial findings for non-cancer conditions such as endometriosis, premature ovarian failure (POF), and uterine fibroids remain controversial and insufficient. However, most current clinical trials for polycystic ovarian syndrome (PCOS) and gynecological malignancies are ongoing phase II–III trials. The pharmacological effects of metformin have been shown to target the insulin-like growth factor (IGF), AMP-activated protein kinase (AMPK), phosphatidylinositol 3-kinase (PI3K)/AKT, MAPK, NF-κB, and other signal transduction pathways, highlighting its potential in the treatment of gynecological disorders. In this review, we discuss the biological impacts of metformin and the mechanisms of action pertinent to the treatment of different gynecological disorders.

## 1 Introduction

Gynecological conditions often determine a woman’s reproductive health and quality of life. However, there is a very high prevalence of gynecological disease worldwide, particularly in developing countries (Bigambo et al., 2022). In China, 45.96% of women suffer from dysmenorrhea, a prevalence lower than that reported in countries such as Ghana (68.1%) and Greece (89.2%). However, the prevalence of ovarian dysfunction in China (11.16%) is notably higher than the global prevalence (3.7%). Most women with gynecological disorders are treated with combination therapies, including surgery, chemotherapy, radiotherapy, and endocrine therapy, which could have serious side effects (He et al., 2024). Thus, it is important to reduce the burden of disease and improve global women’s health through an effective intervention using cheap and widely available drugs (Mousa et al., 2021).

Metformin (N,N-dimethylbiguanide), a standard clinical drug for type 2 diabetes mellitus (T2DM) for over 60 years (Foretz et al., 2023), has been shown to have multiple biological effects beyond its hypoglycemic properties, such as antioxidant, anti-inflammatory, anti-tumor, anti-fibrotic, and antiviral activities (Du et al., 2022; Petrasca et al., 2023; Wang et al., 2023). Recently, metformin has been increasingly acknowledged for its efficacy in the treatment of non-diabetic conditions, such as obesity, cirrhosis of the liver, heart failure, brain damage, and various cancers, including gynecological cancers (Top et al., 2022; Wang et al., 2023). Mechanistically, metformin specifically inhibits mitochondrial respiratory chain complex 1 in a range of tissues, leading to the activation of AMP-activated protein kinase (AMPK) in various tissues, including hepatocytes, muscles, and neurons (Top et al., 2022). Although it is considered an activator of AMPK, evidence supports the involvement of alternative AMPK-independent pathways, suggesting that further research is warranted (Top et al., 2022).

Furthermore, several studies have reported that metformin is effective for the treatment of gynecological disorders, including polycystic ovarian syndrome (PCOS) (Li, Wu et al.), endometriosis, premature ovarian failure (POF), and uterine fibroids. In addition, metformin contributes to a reduction in the risk of gynecologic malignancies, including ovarian, cervical, and endometrial cancers (Shi et al., 2019; Kim et al., 2022; Drevinskaite et al., 2023). Notably, metformin has been established to have treatment efficacy, with several ongoing phase II–III clinical trials in gynecological disorders (Bae-Jump et al., 2020; Garcia-Beltran et al., 2023). This review aims to comprehensively delineate the effects of metformin on gynecological diseases, focusing on its biological mechanisms and therapeutic implications in clinical practice.

## 2 Effects of metformin on PCOS

**FIGURE 1:**
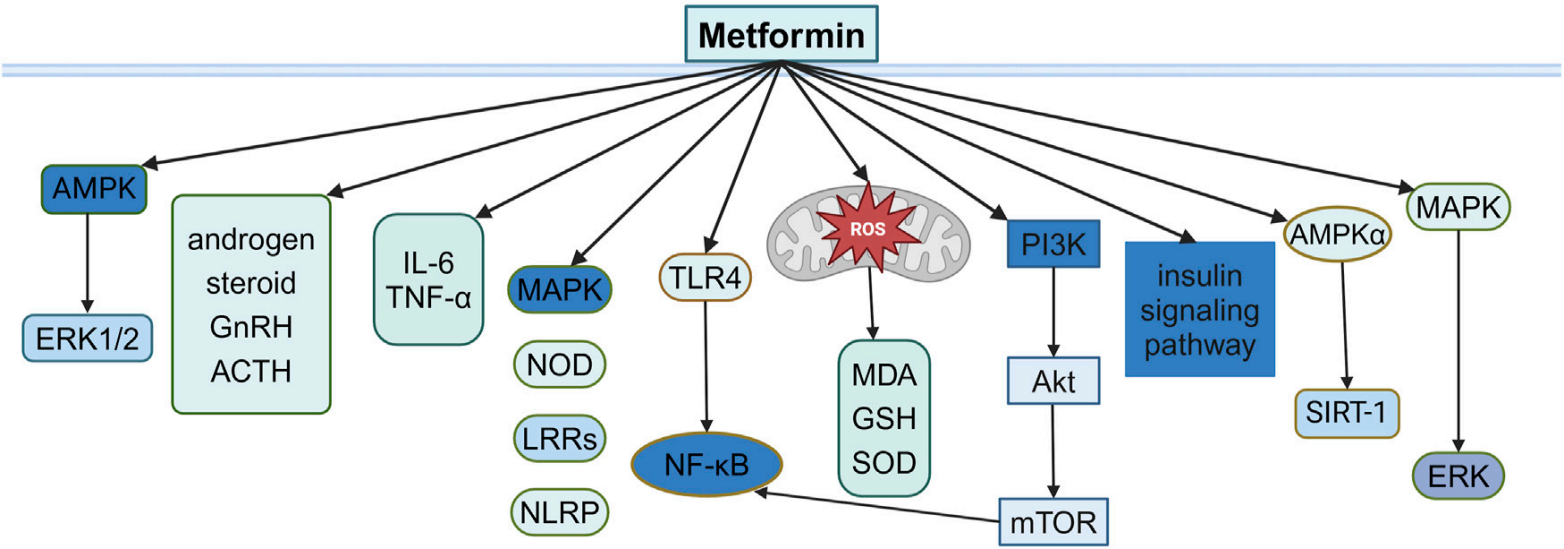
Illustration of the signaling pathway by which metformin affects PCOS.

PCOS is a common disorder of hormonal and metabolic imbalance, characterized by hyperandrogenism, dysfunctional ovulation, and polyfollicular ovaries and accompanied by metabolic abnormalities, such as insulin resistance (IR) and obesity (Zhao et al., 2023), affecting women during their reproductive years, with potential long-term cardiovascular, metabolic, and reproductive health outcomes (Zhu et al., 2022; Anbar et al., 2023). Although the exact cause of PCOS remains unclear, factors such as genetics, oxidative stress, chronic inflammation, and metabolic dysfunction are believed to be involved (Gu et al., 2016; Zhao et al., 2023). Oral contraceptives and hormone therapy are commonly used to manage PCOS, but they carry higher risks of side effects such as venous thromboembolism and reduced bone density, especially in postmenopausal women (Beksinska et al., 2018; Bachrach, 2020). Due to the interconnected nature of IR, obesity, and hyperandrogenism in PCOS, insulin-sensitizing agents, especially metformin, have been frequently studied (Cassar et al., 2016) (Figure 1).

### 2.1 Effects of metformin on IR and hyperandrogenemia in PCOS

PCOS is frequently associated with IR and hyperandrogenemia, affecting 65%–95% of women with the condition (Cassar et al., 2016; Zhao et al., 2023). IR, a defect in insulin signaling that impairs glucose utilization, is a key contributor to metabolic disturbances in PCOS. In addition, sex-hormone-binding globulin (SHBG) is a glycoprotein that transports androgens and estrogens. Androgen excess, a common feature of PCOS, exacerbates IR and is linked to elevated testosterone levels and a decrease in SHBG levels (Xing et al., 2021). Metformin primarily targets IR by enhancing insulin sensitivity, reducing hepatic gluconeogenesis, and increasing muscle glucose uptake (Cassar et al., 2016). It also lowers androgen levels by increasing SHBG, addressing both IR and hyperandrogenemia in PCOS (Diamanti-Kandarakis et al., 1998; Xing et al., 2021).

Metformin also reduces pregnancy-related risks for women with PCOS, such as miscarriage, preeclampsia, and preterm delivery, by improving menstrual regularity and promoting ovulation. A meta-analysis of randomized trials has demonstrated that metformin lowers the incidence of miscarriage (RR = 0.86, 95% CI: 0.67–1.12), preeclampsia (RR = 0.45, 95% CI: 0.24–0.83), and preterm delivery (RR = 0.37, 95% CI: 0.23–0.61) in PCOS patients (Moghetti et al., 2000; Zhu et al., 2022). In addition, metformin treatment greatly reduced gestational diabetes mellitus (GDM) incidence (RR = 0.59, 95% CI 0.43–0.80) compared to placebo, which was attributed to improved β-cell responsiveness and reduced hepatic gluconeogenesis (Yu et al., 2024). Notably, the GDM trial demonstrated that metformin had comparable efficacy to insulin in managing established GDM cases, with additional benefits including attenuated maternal weight gain (mean difference [MD] −2.3 kg, *p* < 0.001) and a 76% reduction in the incidence of preeclampsia (Syngelaki et al., 2016).

Metformin’s effects also extend to cardiovascular risk reduction as it influences key metabolic pathways to improve lipid metabolism and endothelial function, potentially lowering the risk of cardiovascular events (Lan et al., 2015). It has been suggested that metformin displays potential beneficial effects by reducing several cardiovascular dysfunction risk factors, such as body mass index (BMI) (MD: −0.53 kg/m^2^, 95% CI −0.95, −0.12), insulin-resistance (HOMA-IR) (MD:−0.50, 95% CI −0.91, −0.09), triglyceride (MD: −0.11 mmol/L, 95% CI −0.20, −0.02), plasma plasminogen activator inhibitor-1 (Ruth, Day et al.) (MD: −4.99 ng/mL, 95% CI −6.78, −3.21) (Li and Li, 2023), carotid intima-media thickness (CIMT), and flow-mediated dilation (FMD) (Wang et al., 2024) in PCOS patients. Taken together, these data suggest that metformin may help reduce cardiovascular events in PCOS patients. At the molecular level, metformin activates the AMPK or PI3K/mammalian target of rapamycin (mTOR) signaling pathways via oxidative stress, thereby improving glucose and lipid metabolism, and it also contributes to improved endothelial function, which is associated with higher levels of high-density lipoprotein cholesterol (HDL-C); lower levels of low-density lipoprotein cholesterol (LDL-C), triglycerides, and total cholesterol; and reduced risk of cardiovascular events (Anbar et al., 2023; Wang et al., 2024). Animal models reveal that metformin upregulated placental growth factor (PlGF) while suppressing soluble fms-like tyrosine kinase-1 (sFlt-1) production through AMPK-mediated pathways (Tong et al., 2022).

Hyperandrogenism, characterized by elevated androgen levels and typical of PCOS, is often exacerbated by increased ovarian androgen production. Metformin has been shown to directly reduce ovarian androgen levels, independent of its effects on insulin sensitivity (Kurzthaler et al., 2014). This provides a dual therapeutic benefit for both insulin-sensitive and insulin-resistant women with PCOS (la Marca et al., 1999; Vrbikova et al., 2001). Long-term metformin therapy reduces both steroidogenic and metabolic enzyme activities in the ovaries and the adrenal response to adrenocorticotrophin (ACTH), helping mitigate hyperandrogenism in women with PCOS (Lan et al., 2015; LaMoia and Shulman, 2021).

In addition, decreased activity of ERK1/2 with elevated androgens was found in women with PCOS (Guney et al., 2022). *In vitro*, metformin appears to activate ERK1/2 and decrease the activity of CYP19 (P450 aromatase) for estrogen production (Hirsch et al., 2012). The ERK1/2 pathway is activated by luteinizing hormone (LH) in the ovaries, and this activation increases androgen synthesis in theca cells. However, metformin suppresses ERK1/2 activation, reducing androgen levels and improving hormonal balance (Li et al., 2025). In addition, in PCOS rat models, metformin also improves hormonal balance by upregulating the phosphatidylinositol 3-kinase (PI3K) pathway, further demonstrating its multifaceted action on IR and metabolic dysregulation (Guo et al., 2022).

### 2.2 Effects of metformin on inflammation in PCOS

PCOS has been recognized as a low-degree chronic inflammation disease, evidenced by elevated cytokine levels and macrophage infiltration. The inflammatory markers include C-reactive protein (CRP), interleukin-6 (IL-6), interleukin-1β (IL-1β), tumor necrosis factor-alpha (TNF-α), neutrophil-to-lymphocyte ratio (NLR), and platelet-to-lymphocyte ratio (PLR) (Duleba and Dokras, 2012). The increased inflammatory markers are associated with IR-related metabolic dysfunction in PCOS (Gonzalez et al., 2014). Specifically, metformin ameliorates IR and enhances clinical outcomes by reducing the expression of inflammatory markers IL-6 and TNF-α in PCOS in humans (Victor et al., 2015) and animal models (Rabah et al., 2023).

Although the exact etiology of the chronic inflammation process associated with PCOS is not known, it is believed that in adipose tissue, adipocytes and immune cells are the primary sources of cytokine release (Lonardo et al., 2024). Obesity and IR are common risk factors for inflammation, and chronic low-grade inflammation may be more severe in obese and IR women with PCOS (Gonzalez et al., 2014). Macrophages produce inflammatory cytokines that could trigger IR in metabolic tissues. The suppression of macrophages may reduce inflammatory cytokine levels, which are associated with a decrease in IR (Hyun et al., 2013). Furthermore, there is a high prevalence of excess adiposity in PCOS, characterized by enlarged adipocytes and the accumulation of macrophages within adipose tissue (Echiburu et al., 2018).

Previous studies have indicated that metformin exerts anti-inflammatory effects through the inhibition of several pro-inflammatory signaling pathways, such as MAPK, nucleotide-binding oligomerization domain (NOD)-like receptors, leucine-rich repeats (LRRs), and NLR family pyrin domain containing 3 (NLRP3) inflammasome (Jia et al., 2020; Jin et al., 2022). NF-κB is a key factor in determining the inflammatory condition in PCOS, and the activation of NF-κB leads to increased inflammation. Metformin suppresses the PI3K-AKT-NF-κB signaling pathway and reduces the expression of inflammatory genes in PCOS-like rats (Zhang et al., 2017). In addition, Toll-like receptors (TLRs) mediate inflammatory responses associated with increased interleukin accumulation and contribute to the pathogenesis of PCOS (Gu et al., 2016). Metformin treatment effectively attenuates the release of inflammatory cytokines in endometrial tissues via the TLR4/NF-κB signaling pathway (Hu et al., 2021). Additionally, metformin reduces inflammatory cytokines by decreasing leukocyte–endothelium interactions in PCOS (Victor et al., 2015).

### 2.3 Effects of metformin on oxidative stress in PCOS

Oxidative stress, an imbalance between oxidants and antioxidants, is caused by excessive reactive oxygen species (ROS) production. It has been suggested that ROS levels are elevated in women with PCOS (Duleba and Dokras, 2012). Mitochondrial dysfunction is the main source of ROS, and it is especially important for ovarian function and metabolic activity (Rabah et al., 2023; Wu et al., 2023; Chen et al., 2024). ROS may directly lead to IR and hyperandrogenism through the p47^phox^ (a NADPH oxidase subunit) component, which is related to the response of immune cells within adipose tissue in women with PCOS (Gonzalez et al., 2019). On the other hand, ROS activates the NF-κB signaling pathway, which stimulates an inflammatory response that further increases IR and hyperandrogenism (Rudnicka et al., 2022). IR in the skeletal muscle of women with PCOS is associated with reduced expression of genes involved in mitochondrial oxidative metabolism and reduced expression of peroxisome proliferator-activated receptor γ coactivator α (PGC-1α), which interacts with AMPK or proliferator-activated receptor γ (PPARγ) and controls proteins involved in the regulation of mitochondrial function and metabolism, including oxidative phosphorylation (*OXPHOS*) gene and mitochondrial DNA (mtDNA) replication (Skov et al., 2007; Abu Shelbayeh et al., 2023).

Metformin, with its antioxidant properties, reduces ROS levels and improves mitochondrial function and insulin sensitivity (Udono and Nishida, 2022). In clinical trials, metformin therapy in PCOS women improves oxidative stress markers, including a decrease in malondialdehyde (MDA) levels and an increase in glutathione (GSH) levels and superoxide dismutase (SOD) enzyme activity (Xu et al., 2022). Moreover, metformin improves mitochondrial function and contributes to helping restore hormonal balance and insulin sensitivity in PCOS patients (Rabah et al., 2023). The PI3K/AKT/mTOR signaling pathway has been demonstrated to be the most disturbed pathway in ovarian granulosa cells (GCs) under oxidative stress and in PCOS (Abuelezz et al., 2020). Metformin decreases ROS levels, resulting in reduced autophagy via the PI3K/AKT/mTOR signaling pathway in H_2_O_2_-induced GCs and a PCOS rat model (Xu et al., 2022). Studies in the PCOS–IR rat model demonstrate that metformin reduces ROS levels, resulting in improved mitochondrial function and insulin sensitivity and upregulated PI3K and AKT gene expression (Rabah et al., 2023).

## 3 Effects of metformin on endometriosis

**FIGURE 2:**
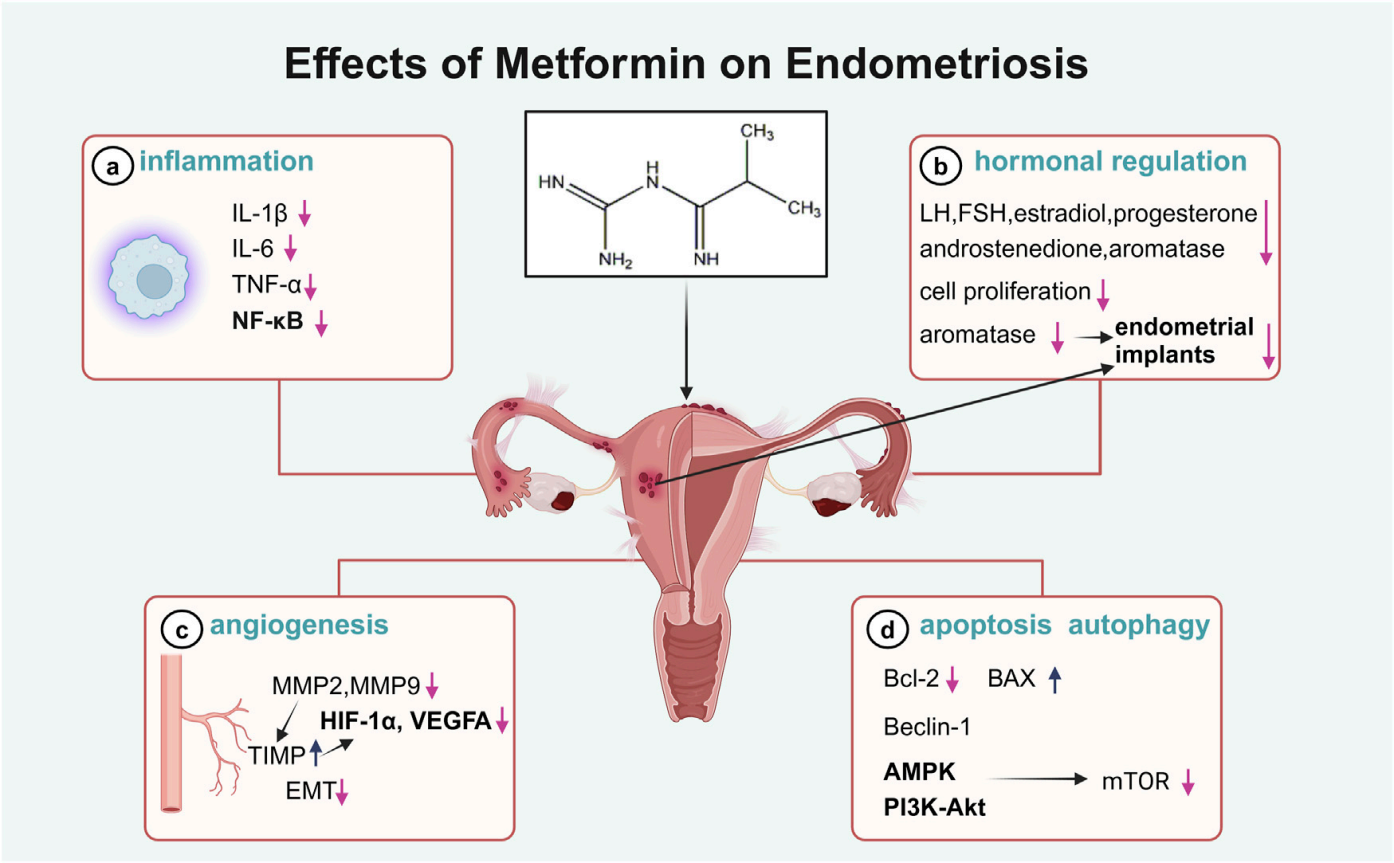
Illustration of the effects of metformin in endometriosis.

Endometriosis is a common gynecological condition affecting 5%–15% of women of reproductive age worldwide, leading to pain, infertility, and systemic inflammation (Taylor et al., 2021). The condition is characterized by altered lipid metabolism in the liver and adipose tissue, contributing to elevated levels of inflammatory markers such as IL-6, TNF-α, and CRP (Taylor et al., 2021). The recommended first-line treatments for endometriosis, such as nonsteroidal anti-inflammatory drugs (NSAIDs) and hormonal therapies, are often poorly tolerated due to their side effects and limited efficacy in reducing ectopic lesions (Brown et al., 2017; Peitsidis et al., 2023). Although studies in women with endometriosis treated with metformin are scarce, it has been demonstrated that metformin may avoid the main side-effects of other treatments and exert anti-inflammatory, anti-proliferative, antioxidative, and anti-angiogenic effects on endometriosis, suggesting its potential role in the management of endometriosis (Figure 2).

### 3.1 Effects of metformin on hormonal regulation in endometriosis

Endometriosis is a hormone-dependent condition with estrogen dependency and progesterone resistance. Elevated estrogen production and marked progesterone resistance are the key events that promote the ectopic implantation of endometrial cells (Vannuccini et al., 2022). Metformin modulates steroid hormone levels, inhibiting the secretion of follicle-stimulating hormone (FSH), LH, estradiol, progesterone, and androstenedione in ovarian GCs (Rice et al., 2013). Endometriosis is characterized by the ectopic growth of endometrial stromal cells and glands. Metformin has been shown to have inhibitory effects on the growth of progesterone-resistant endometrial epithelial cells and peritoneal adhesions in animal models (Zhuo et al., 2016; Hu et al., 2018).

Importantly, aromatase is a member of the cytochrome P450 (CYP) superfamily and induces the aromatization of androgens into estrogens. High levels of steroidogenic acute regulatory protein and aromatase are expressed in endometriotic stromal cells (ESCs), contributing to the growth of endometrial implants (Xu et al., 2014). The inhibition of aromatase is one of the targets of available and emerging drugs for endometriosis. Several findings have shown that metformin could suppress aromatase activity by inhibiting prostaglandin E2 (PGE2)-induced CYP19A1 and StAR expression in endometriotic stromal cells; thus, it appears to be effective in the medical management of endometriosis (Xu et al., 2014; Zhou et al., 2015).

### 3.2 Effects of metformin on inflammation in endometriosis

In patients with endometriosis, aberrant inflammatory responses are observed, with elevated levels of pro-inflammatory cytokines, including TNF-α, IL-1β, and IL-6, found in the serum, peritoneal fluid, and ectopic lesions (Zhou et al., 2019; Sapmaz et al., 2022). Metformin therapy may relieve the clinical symptoms of endometriosis (pelvic pain and menstrual disorders), accompanied by a reduction in the levels of inflammatory cytokines in the serum and peritoneal fluid in women with endometriosis due to its anti-inflammatory effects (Omer, 2016).

NF-κB has been extensively reported to play a role in the progression of endometriosis, particularly in lesion development, cell proliferation, and angiogenesis. In patients with endometriosis, activation of NF-κB is found in endometrial cells, macrophages, and peritoneal fluid (Liu et al., 2022), contributing to the proliferation of endometriotic cells (Wang et al., 2022) and the polarization of peritoneal macrophages (Lousse et al., 2008). In addition to inflammatory cells, other cell types, such as stromal cells, fibroblasts, and endothelial cells, could produce inflammatory cytokines and contribute to pro-inflammatory effects (Mohebbi et al., 2022). Studies on endometriosis have shown that metformin exerts potent anti-NF-κB effects, alleviating disease progression, and it has demonstrated the potential to target pro-inflammatory molecules in endometriotic cells (Takemura et al., 2007), macrophages, and vascular smooth muscle cells *in vitro* (Isoda et al., 2006), in obese mice (Hyun et al., 2013), and in rat models of endometriosis (Jamali et al., 2021; Sapmaz et al., 2022). These findings indicate the significant therapeutic potential of metformin in endometriosis management by targeting inflammatory pathways.

### 3.3 Effects of metformin on angiogenesis in endometriosis

Angiogenesis is fundamental to the growth of endometriotic tissue, which starts with the destabilization of the pre-existing vasculature, and is driven by the release of pro-angiogenic factors and the degradation of the extracellular matrix (ECM). Matrix metalloproteinases (MMPs), regulated by their endogenous inhibitors—tissue inhibitors of metalloproteinases (TIMPs) (Ruth, Day et al.) (Ruth, Day et al.)—are critical for ECM breakdown and the promotion of angiogenesis (Zafrakas et al., 2020). Higher levels of vascular endothelial growth factor (VEGF), MMPs, and reduced TIMPs have been observed in endometriotic lesions, serum, and peritoneal fluid from endometriosis patients (Zafrakas et al., 2020; Li et al., 2021) and in animal models (Lu et al., 2006; Maoga et al., 2023). Suppression of MMPs has been reported to inhibit the establishment of ectopic lesions derived from human endometrium in nude mice (Bruner et al., 1997). The potential effect of metformin on angiogenesis in endometriosis may be mediated through the regulation of MMP activity. Metformin reduces the expression of MMP2 and MMP9 and enhances TIMP expression, accompanied by increased expression of angiogenesis-related genes, such as *HIF-1α* and *VEGFA*, in human ectopic endometrial cells (Yari et al., 2021) and endometriotic implants of endometriosis rat models (Yilmaz et al., 2010; Cheng et al., 2022).

Furthermore, vascular dysfunction is associated with the upregulation of endothelin-1 (ET-1) and reduced endothelial nitric oxide synthase (eNOS) production and contributes to endothelial damage in endometriosis (Wu et al., 2003). In a mouse model of endometriosis, daily metformin for 3 months led to a significant reduction in ET-1 expression, an increase in eNOS expression, and diminished endometriosis-associated endothelial dysfunction (Martins et al., 2022; Yang et al., 2024). Studies involving metformin administration in women with endometriosis are scarce. However, it has been demonstrated that metformin treatment has an impact on the angiogenic, inflammatory, and ECM-related genes, suggesting its potential role in the regulation of angiogenesis in endometriosis.

### 3.4 Effects of metformin on apoptosis and autophagy in endometriosis

Endometriosis is characterized by enhanced proliferation and diminished apoptosis of endometrial cells. Endometriotic endometrial cells show an overexpression of anti-apoptotic genes such as *Bcl-2* and insufficient expression of pro-apoptotic factors such as Bax (Braun et al., 2007), and an apoptosis-inducing agent is considered to be a promising therapeutic strategy for endometriosis. Apoptosis is controlled by NF-κB transcription factors in a wide range of cell types, including endometrial cells. Metformin increases the apoptotic index by modulating the Bcl-2/Bax ratio in endometrial cells via NF-κB activation (Sapmaz et al., 2022; Wang et al., 2022).

Autophagy has been considered an efficient regulator of apoptosis (Kobayashi et al., 2024). Beclin-1 is an important autophagic factor and can interact with Bcl-2. The levels of the Beclin-1 and Bcl-2 proteins in endometriotic lesions suggest the existence of an autophagy and apoptosis dysfunction in endometriosis in humans (Li et al., 2021) and induced rodent models (Li et al., 2023). The autophagy process is associated with the PI3K/AKT/mTOR signaling pathway and downstream target molecules, including autophagy-related proteins (Kobayashi et al., 2024). mTOR is the main suppressor of autophagy (Kim and Guan, 2015; Jamali et al., 2021), and metformin has a strong inhibitory effect on mTOR by stimulating AMPK or NF-κB (Lu et al., 2021; Saber and El-Kader, 2021). In *in vitro* cultured human endometrial stromal cells (HESCs), overexpression of HIF-1α results in enhanced cell migration, invasion ability, and autophagy, and Beclin-1 and light chain 3 (LC3) are upregulated (Liu et al., 2017; Zhang et al., 2024). In endometriotic lesions of mice, the PI3K/AKT/mTOR pathway was activated, and metformin administration was associated with the enhanced autophagy process through mTOR inhibition (Jamali et al., 2021). These observations suggest an anti-endometriotic effect of metformin through autophagy-related pathways. To date, clinical studies on metformin’s effects on autophagy have been conducted in patients with T2DM, PCOS, and cancer (Lu et al., 2021). Further investigation is needed to determine the pharmacological effects of metformin on endometriosis via autophagy.

## 4 Effects of metformin on POF

**FIGURE 3:**
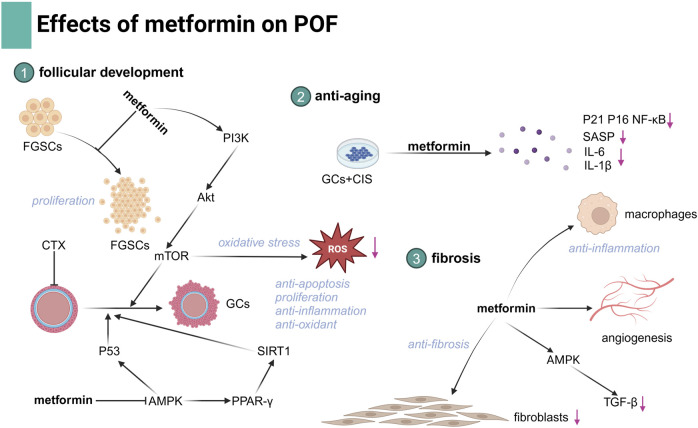
Illustration of the effects of metformin in POF.

POF is an important cause of infertility characterized by the functional decline of the ovary. POF affects 3%–7% of reproductive-aged women, particularly those undergoing chemotherapy (Golezar et al., 2019). During folliculogenesis, GCs play a crucial role in supporting oocyte growth and maturation. Dysfunction of GCs is a key factor in the development of POF (Wu et al., 2023). The etiologies of POF are heterogeneous and multifactorial, and traditional therapies have limited effectiveness, benefiting only a small proportion of patients. Metformin, with its low cost and established safety profile, has demonstrated promising effects in improving ovarian function. It exerts anti-inflammatory, antioxidant, and anti-apoptotic effects, which contribute to the restoration of ovarian hormonal function. These effects suggest that metformin could be a potential therapeutic option for fertility preservation in POF, offering a novel approach for managing this condition in the near future. Despite promising results, further research is needed to optimize metformin’s efficacy in POF treatment (Figure 3).

### 4.1 Effects of metformin on follicular development in POF

POF occurs due to the exhaustion of a number of ovarian follicle-associated endocrine dysfunctions (Wu et al., 2023). Female germline stem cells (FGSCs) are stem cells that can differentiate into mature oocytes and remodel ovarian function. Some studies have shown that replenishing exhausted FGSCs is a promising approach to restore ovarian function (Hong et al., 2022). It has been demonstrated that metformin treatment can promote FGSC proliferation and facilitate follicular maturation, thus enhancing ovarian reserve capabilities (Chen et al., 2023).

There exists abnormal GC damage in the ovary of POF patients (Lin et al., 2022). GCs play a crucial role in follicle initiation and development, and abnormal GC proliferation or apoptosis is an important factor causing POF. Steroid hormone secretion is one of the key functions of GCs. *In vitro* studies using cultured ovarian cells have demonstrated a direct metformin effect on ovarian steroidogenesis (Kurzthaler et al., 2014). Moreover, cyclophosphamide (CTX) induces increased apoptosis and reduced cellular proliferation of GCs, and metformin can exert anti-apoptotic and cell proliferative effects against chemotoxicity via the AMPK-dependent p53 signaling pathway (Huang et al., 2021).

Recent studies using a chemotherapy- and D-galactose-induced POF mouse model have reinforced the beneficial effects of metformin in enhancing ovarian function (Du et al., 2022; Ellibishy et al., 2024). Cisplatin exposure directly induces follicle loss, while metformin treatment encourages the resumption of follicular growth and development in the ovaries, showing that primordial follicles and growing follicles are largely restored and atretic follicles are significantly reduced in cisplatin-treated ovaries. In the CTX- and D-galactose-exposed mouse model, the use of metformin is associated with improved serum hormonal levels and follicle numbers, exhibiting mTOR-inhibitory and anti-apoptotic effects (Huang et al., 2021). Additionally, the effect of metformin may also be achieved through an improvement in anti-inflammatory and antioxidant properties on GCs, and the activation of the PI3K/AKT/mTOR or AMPK/PPAR-γ/SIRT1 signaling pathway is involved (Ellibishy et al., 2024; Yang et al., 2024).

In addition to the hormonal and metabolic dysfunctions commonly associated with POF, genetic factors also play a significant role in its pathogenesis. Genetic mutations, such as in the *FMR1* gene (fragile X messenger ribonucleoprotein 1), have been linked to genetically induced POF (Ojavee et al., 2023). There is also some evidence that metformin may be effective in improving aberrant behavior and correcting electrophysiological abnormalities in patients with fragile X syndrome, but no clinical trials have tested its effects on ovary function in these patients (Proteau-Lemieux et al., 2021). Further research is needed to evaluate the therapeutic limitations and potential effects of metformin in patients with genetically induced POF. Furthermore, genetic mutations causing POF may impair follicular development and function independently of insulin or metabolic regulation, and metformin may aid in managing secondary metabolic complications.

### 4.2 Metformin as an anti-aging agent in POF

Metformin has been identified as an anti-aging agent in a clinical trial-TAME (targeting aging by metformin) and can relieve age-associated pathologies (Kulkarni et al., 2020). Beyond its use in cardiovascular diseases, cancer, osteoarthritis, Alzheimer’s disease, and obesity, metformin could alleviate aging and age-related phenotypes by suppressing senescence through mechanisms such as redox balance, autophagy, and immune and inflammation response in the ovary (Qin et al., 2019; Lu et al., 2021; Landry et al., 2022; Yang et al., 2024).

*In vitro* cultures of cisplatin-treated GCs to which metformin was added have displayed a significant reduction in the expression of senescence markers (p21, p16, and NF-κB) and senescence-associated secretory phenotype (SASP) markers, IL-6 and IL-1β, and in a number of senescence-associated beta-galactosidase (SAβ-gal)-staining cells, and ultimately, improved functional and structural changes occurred in cisplatin-induced POF mice (Du et al., 2022). Oxidative stress promoting aging in GCs was considered the primary pathogenesis of POF (Lin et al., 2022). Metformin could attenuate H_2_O_2_-induced oxidative stress and further decrease excessive autophagy caused by oxidative stress via the PI3K/AKT/mTOR pathway in rat GCs (Xu et al., 2022). Additionally, metformin significantly reduces macrophage-induced ROS accumulation and senescence in primary GCs by inducing the expression of the AMPK pathway and protects against CTX-induced POF (Yang et al., 2024).

### 4.3 Effects of metformin on fibrosis in POF

POF is associated with significant changes in the structural organization of collagen, characterized by excessive deposition of the ECM, resulting in ovarian fibrosis (Pellicer et al., 2023). Metformin has been demonstrated to effectively treat and prevent fibrosis in various organs, including the lungs (Cheng et al., 2021), kidneys (Wang et al., 2016), liver (Pinyopornpanish et al., 2021), and heart (Li et al., 2023). In the fibrotic ovary, several studies have demonstrated that metformin attenuates ovarian fibrosis through the modulation of immune cells, fibroblasts, and angiogenesis, and pro-fibrotic and inflammation signaling pathways were involved, particularly in aged or high-fat diet-induce mouse models (McCloskey et al., 2020; Landry et al., 2022; Velazquez et al., 2023).

Fibrosis is associated with a pro-inflammatory cascade characterized by enhanced M1-like macrophages, reduced M2-like macrophage infiltration, and elevated pro-inflammatory chemokines, which are believed to damage GCs in aged and POF ovaries (McCloskey et al., 2020; Yang et al., 2024). Transforming growth factor-β (TGF-β) is known to promote fibrosis and contribute to POF (Persani et al., 2011), and collagen content and fibroblast proliferation are significantly reduced, which is related to AMPK-mediated suppression of TGF-β production after metformin administration (McCloskey et al., 2020). In addition, mitochondrial dysfunction in stromal cells is the key causal factor triggering fibrosis-induced ovarian decline, and metformin’s reversal of ovarian fibrosis converges on related mitochondrial metabolic pathways that are upstream of oxidative stress and inflammation (Umehara et al., 2022). Ovarian fibrosis has been established in human chemotherapy-induced POF, but the effect of metformin on POF-associated murine ovarian fibrosis has yet to be extended to human POF (Meirow et al., 2007).

## 5 Effects of metformin on uterine fibroids

Uterine leiomyomata or fibroids are extremely common benign gynecological tumors that occur in 80% of reproductive-aged women, and they are commonly associated with heavy menstrual bleeding (Moravek and Bulun, 2015; George, 2023). Recent studies have suggested that the drug metformin might exert a beneficial effect on the management of uterine fibroids through mechanisms including anti-proliferative actions, promotion of apoptosis, and angiogenesis inhibition (Li et al., 2013; Tadakawa et al., 2015).

A population-based retrospective cohort study supports a reduced risk of uterine fibroids associated with metformin use in Taiwanese female patients with T2DM (Tseng, 2019). *In vitro*, metformin has anti-tumor properties, inhibiting proliferation and inducing apoptosis on uterine leiomyoma cells via the activation of AMPK, followed by the inhibition of the mTOR pathway (Li et al., 2013; Tadakawa et al., 2015). Metformin has also been shown to inhibit the proliferation of leiomyoma cells to reduce tumor size in clinical trials (Wang et al., 2019).

Additionally, fibroid growth is primarily dependent on the levels of circulating estrogen and the regulation of estrogen signaling (Borahay et al., 2017). 17β-hydroxysteroid dehydrogenase (17β-HSD) and aromatase are the major enzymes catalyzing the conversion of androstenedione to estrone and are found to be overexpressed in fibroid tissue than in normal myometrium, and this suggests that fibroids convert circulating androstenedione into estrone *in situ* (Sumitani et al., 2000). In contrast, metformin could suppress the expression of HSD and aromatase (P450 arom) in uterine fibroid cells and reduce estrogen synthesis, thereby inhibiting the growth of fibroids (Wang et al., 2019).

Angiogenesis and vascularization have been regarded as crucial factors controlling the growth of tumors. Multiple angiogenic factors, such as VEGF and its receptors, are overexpressed in leiomyoma tissue compared to the adjacent myometrium (Di Tommaso et al., 2013). In addition, metformin suppresses VEGF expression through the mTORC1/HIF-1α pathway, further indicating its anti-angiogenic properties in uterine fibroids (Tadakawa et al., 2015).

## 6 Effects of metformin on gynecologic malignancies

### 6.1 Ovarian cancer

Based on population-based retrospective studies and meta-analysis, there is a markedly reduced risk of ovarian cancer incidence associated with metformin use by patients with T2DM (Tseng, 2015; Shi et al., 2019). Additionally, in clinical trials, metformin treatment enhances the survival of ovarian cancer patients (Kumar et al., 2013). The anti-tumor effects of metformin on ovarian cancer are attributed primarily to its ability to inhibit cancer cell proliferation, enhance the sensitivity of cancer cells to chemotherapeutic agents, modulate the cell cycle, and promote apoptosis in cancerous cells.

Extensive research has demonstrated that metformin treatment can suppress the proliferation, chemoresistance, and metastasis of ovarian cancer cells (Rattan et al., 2011; Min et al., 2020). The anti-proliferation effect of metformin may involve p53 protein ubiquitination or the NF-κB signaling pathway (Min et al., 2020; Zheng et al., 2022). Moreover, metformin induces cell cycle arrest and apoptosis through the Bcl-2-family of proteins in primary human ovarian carcinoma cells, isolated from ascitic fluid or omental metastases (Liu et al., 2018). In addition, metformin significantly inhibits angiogenesis through downregulated VEGF activity induced by the IL-6/STAT3 or AMPK/mTOR signaling pathways (Rattan et al., 2011; Yang et al., 2021).

Furthermore, metformin enhances the anti-tumor efficacy by diminishing tumor resistance *in vivo* and *in vitro*. Several studies suggest that metformin induces growth inhibition and shows synergistic effects in overcoming chemoresistance, especially in cisplatin-resistant ovarian cancer cells (Lee et al., 2019), and the chemosensitizing effect of metformin seems to be dependent on p53 function (Han et al., 2019). Increased Bcl-2-protein family-dependent apoptosis was also linked to metformin’s chemosensitizing effects in ovarian cancer (Yasmeen et al., 2011). In cisplatin-resistant ovarian cancer patient-derived xenograft models, *in vivo* treatment with metformin partially reversed platinum resistance (Ricci et al., 2019). Furthermore, metformin sensitization of drug-resistant ovarian cancer to chemotherapeutic agents is possibly through the induction of autophagy (Yang et al., 2019). These clinical and laboratory studies support the essential role of metformin in the development and growth of ovarian cancer, highlighting its potential as a therapeutic agent against ovarian cancer.

### 6.2 Cervical cancer

Retrospective cohort studies demonstrated that metformin use in patients with diagnosed T2DM was associated with a lower risk of cervical cancer (Kim et al., 2022). Although there is one study demonstrating no association between metformin use and the survival outcome of women with cervical cancer (Takiuchi et al., 2017), several studies revealed that metformin treatment decreased cervical cancer-specific and overall mortality in older women with T2DM (Han et al., 2016) and reduced the recurrence rate in patients with T2DM (Hanprasertpong et al., 2017).

It has been reported that metformin inhibits cell viability, migration, and metastasis and induces cell-cycle arrest, autophagy, and apoptosis in cervical cancer cell lines and cervical cancer xenografts (Cheng and Hao, 2016; Xia et al., 2018). These actions of metformin are mediated via various mechanisms, such as PI3K/AKT/mTOR inhibition and AMPK pathway activation (Xia et al., 2020; Chen et al., 2021). Metformin has been reported to inhibit heme oxygenase-1 (HO-1) expression in cervical cancer HeLa cells, contributing to the regulation of angiogenesis and cell proliferation (Do et al., 2013).

By targeting the PI3K/AKT and p53 pathways, metformin could also enhance NK cell cytotoxicity and may be used as an immunopotentiator for combination therapy with immunotherapy (Xia et al., 2020). By targeting mitochondria, metformin suppresses the malignant phenotype and metastatic potential of cervical cancer cells due to the modulation of mitochondrial respiration and glucose metabolism through the citric acid cycle (Tyszka-Czochara et al., 2017). Furthermore, metformin is found to amplify chemotherapy-induced AMPK activation and sensitize cervical cancer cells to the action of cisplatin (Tyszka-Czochara et al., 2017). These results suggest that metformin exerted a synergistic action in chemotherapy-based strategies for cervical cancer treatment.

### 6.3 Endometrial carcinoma

The incidence of endometrial cancer is increasing in parallel with the rising prevalence of metabolic syndrome, obesity, and T2DM (Drevinskaite et al., 2023). Metformin is used as a first-line therapy to target risk factors that contribute to endometrial cancer, such as PCOS and obesity, due to its effects on IR and glucose metabolism. Although epidemiologic studies evaluating the relationship between metformin use and endometrial cancer risk are conflicting (Tang et al., 2017; Drevinskaite et al., 2023), other studies demonstrated that metformin could improve endometrial cancer outcomes, reducing mortality risk and prolonging overall survival of endometrial cancer patients (Xie et al., 2024).

Endometrial cancers are traditionally classified as type 1 (estrogen-dependent) and type 2 (estrogen-independent) categories, and type 1 cancers, which are usually endometrioid in histology, account for ∼90% of all endometrial cancer cases . Endometrioid endometrial cancer is driven by obesity and IR (Kitson et al., 2019). Metformin improves IR, and evidence has suggested that its use is associated with improved glucose metabolism in women with endometrial cancer (Davis et al., 2018). In endometrial cancer cell cultures, metformin treatment reduces the secretion of insulin-like growth factor (IGF-1) and downregulates the expression of the insulin receptor and IGF-1R (Sarfstein et al., 2013). In addition to IGF pathways, anti-tumor effects on PI3K/AKT/mTOR and MAPK/ERK pathways with metformin exposure were observed, leading to the upregulation of markers of cell cycle arrest, apoptosis, and autophagy and downregulation of markers associated with senescence and the inhibition of cell migration (Hanna et al., 2012; Lee et al., 2018; Cao et al., 2019; Ruiz-Mitjana et al., 2023).

**FIGURE 4:**
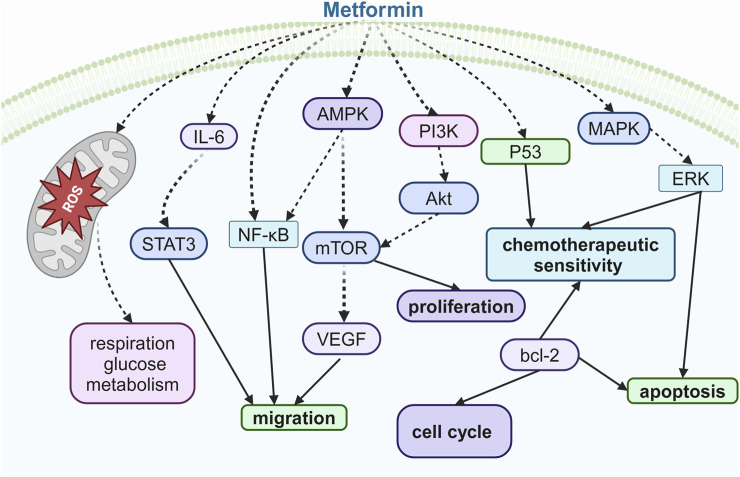
Illustration of the signaling pathway by which metformin affects ovarian, cervical, and endometrial cancers.

More importantly, metformin exhibits properties of sensitizing cancer cell to chemotherapy and hormone therapies. Progesterone is a key hormone in the endometrium that opposes estrogen-driven growth, and insufficient progesterone action strikingly increases the risk of endometrial cancer (Alhujaily et al., 2021). Glyoxalase I (GLOI) and ten–eleven translocation 1 (TET1), along with 5-hydroxymethylcytosine (5-hmC), contribute to progestin resistance and chemotherapeutic resistance in endometrial cancer (Lv et al., 2017; Alhujaily et al., 2021; Liu et al., 2021). It has been reported that metformin treatment increased the sensitivity of endometrial cells to cisplatin and paclitaxel, an effect that was associated with reduced levels of GLOI expression (Hanna et al., 2012). Additionally, metformin sensitizes progestin and exerts anti-estrogen capacity in endometrial cancer through the TET1-5hmC-GLOI signaling pathway (Jiang et al., 2019). Isocitrate dehydrogenase 1 (IDH1) is an enzyme that catalyzes isocitrate to produce α-ketoglutarate (α-KG), a substrate of TET1, which controls TET1-mediated progestin- and chemotherapy-resistant genes in endometrial cancer (Li et al., 2024). Metformin could mediate the induction of chemosensitivity resulting from the downregulation of Nrf2, leading to the inhibition of IDH1-ɑ-KG-TET1-Nrf2 signaling (Bai et al., 2018; Jiang et al., 2019). Overall, the efficacy of metformin use in endometrial cancer is promising. However, in one multi-center, randomized phase-III trial, short-term treatment with metformin did not reduce tumor proliferation in women with endometrioid endometrial cancer (Kitson et al., 2019); therefore, clinical trials to confirm the effects of metformin on endometrial cancer are warranted (Figure 4).

## 7 Conclusion

Many *in vitro*, *in vivo*, and pre-clinical studies have demonstrated various effects of metformin, such as anti-diabetic, antioxidant, anti-inflammatory, anti-tumor, and cardioprotective effects in gynecological disorders, which are mainly due to the effects on the IGF system and several intracellular signaling transduction pathways, such as AMPK, PI3K/AKT/mTOR, and MAPK/ERK pathways. Although clinical studies confirm the efficacy of metformin in treating conditions such as PCOS, the evidence for its role in endometriosis, POF, myomas, and gynecological cancers is limited. In terms of gynecological cancers, while some clinical trials have shown mixed results regarding metformin’s anti-proliferative activity, there is emerging evidence suggesting its role in enhancing chemotherapy sensitivity and improving insulin sensitivity in cancer patients. However, the specific contribution of metformin in the treatment of endometrial cancer remains unclear, and further research is needed to clarify its potential as an adjunct to existing cancer therapies. In conclusion, while metformin has shown promise in several gynecological disorders, more targeted clinical trials are necessary to establish its role in endometriosis, POF, myomas, and gynecological malignancies.

## References

[B1] Abuelezz N. Z. Shabana M. E. Abdel-Mageed H. M. Rashed L. Morcos G. N. B. (2020). Nanocurcumin alleviates insulin resistance and pancreatic deficits in polycystic ovary syndrome rats: insights on PI3K/AkT/mTOR and TNF-alpha modulations. Life Sci. 256, 118003. 10.1016/j.lfs.2020.118003 32589998

[B2] Abu Shelbayeh O. Arroum T. Morris S. Busch K. B. (2023). PGC-1α is a Master regulator of mitochondrial lifecycle and ROS stress response. Antioxidants (Basel) 12 (5), 1075. 10.3390/antiox12051075 37237941 PMC10215733

[B3] Alhujaily M. Abbas H. Xue M. Z. de la Fuente A. Rabbani N. Thornalley P. J. (2021). Studies of glyoxalase 1-linked Multidrug resistance reveal Glycolysis-derived reactive metabolite, Methylglyoxal, is a common contributor in cancer chemotherapy targeting the Spliceosome. Front. Oncol. 11, 748698. 10.3389/fonc.2021.748698 34790575 PMC8591171

[B4] Anbar H. S. Vahora N. Y. Shah H. L. Azam M. M. Islam T. Hersi F. (2023). Promising drug candidates for the treatment of polycystic ovary syndrome (PCOS) as alternatives to the classical medication metformin. Eur. J. Pharmacol. 960, 176119. 10.1016/j.ejphar.2023.176119 37852569

[B5] Bachrach L. K. (2020). Hormonal contraception and bone health in Adolescents. Front. Endocrinol. (Lausanne) 11, 603. 10.3389/fendo.2020.00603 32973688 PMC7472551

[B6] Bae-Jump V. L. Sill M. Gehrig P. A. Moxley K. Hagemann A. R. Waggoner S. E. (2020). A randomized phase II/III study of paclitaxel/carboplatin/metformin versus paclitaxel/carboplatin/placebo as initial therapy for measurable stage III or IVA, stage IVB, or recurrent endometrial cancer: an NRG Oncology/GOG study. Gynecol. Oncol. 159, 7. 10.1016/j.ygyno.2020.06.013 PMC12368636 40056832

[B7] Bai M. Z. Yang L. L. Liao H. Liang X. Y. Xie B. Y. Xiong J. (2018). Metformin sensitizes endometrial cancer cells to chemotherapy through IDH1-induced Nrf2 expression via an epigenetic mechanism. Oncogene 37 (42), 5666–5681. 10.1038/s41388-018-0360-7 29921847

[B8] Beksinska M. E. Kleinschmidt I. Smit J. A. (2018). Bone mineral density in midlife long-term users of hormonal contraception in South Africa: relationship with obesity and menopausal status. Womens Midlife Health 4, 6. 10.1186/s40695-018-0035-0 30766716 PMC6297953

[B9] Bigambo F. M. Wang D. Zhang Y. Mzava S. M. Dai R. Wang X. (2022). Current situation of menstruation and gynecological diseases prevalence among Chinese women: a cross-sectional study. BMC Womens Health 22 (1), 270. 10.1186/s12905-022-01860-5 35787274 PMC9254498

[B10] Borahay M. A. Asoglu M. R. Mas A. Adam S. Kilic G. S. Al-Hendy A. (2017). Estrogen receptors and signaling in fibroids: role in Pathobiology and therapeutic implications. Reprod. Sci. 24 (9), 1235–1244. 10.1177/1933719116678686 27872195 PMC6344829

[B11] Braun D. P. Ding J. Shaheen F. Willey J. C. Rana N. Dmowski W. P. (2007). Quantitative expression of apoptosis-regulating genes in endometrium from women with and without endometriosis. Fertil. Steril. 87 (2), 263–268. 10.1016/j.fertnstert.2006.06.026 17094974

[B12] Brown J. Crawford T. J. Allen C. Hopewell S. Prentice A. (2017). Nonsteroidal anti-inflammatory drugs for pain in women with endometriosis. Cochrane Database Syst. Rev. 1 (1), CD004753. 10.1002/14651858.CD004753.pub4 28114727 PMC6464974

[B13] Bruner K. L. Matrisian L. M. Rodgers W. H. Gorstein F. Osteen K. G. (1997). Suppression of matrix metalloproteinases inhibits establishment of ectopic lesions by human endometrium in nude mice. J. Clin. Invest. 99 (12), 2851–2857. 10.1172/JCI119478 9185507 PMC508135

[B14] Cao C. Zhou J. Y. Xie S. W. Guo X. J. Li G. T. Gong Y. J. (2019). Metformin enhances Nomegestrol Acetate suppressing growth of endometrial cancer cells and may correlate to downregulating mTOR activity *in vitro* and *in vivo* . Int. J. Mol. Sci. 20 (13), 3308. 10.3390/ijms20133308 31284427 PMC6650946

[B15] Cassar S. Misso M. L. Hopkins W. G. Shaw C. S. Teede H. J. Stepto N. K. (2016). Insulin resistance in polycystic ovary syndrome: a systematic review and meta-analysis of euglycaemic-hyperinsulinaemic clamp studies. Hum. Reprod. 31 (11), 2619–2631. 10.1093/humrep/dew243 27907900

[B16] Chen H. Nie P. Li J. Wu Y. Yao B. Yang Y. (2024). Machine learning models in evaluating the malignancy risk of ovarian tumors: a comparative study. J. Ovarian Res. 17 (1), 219. 10.1186/s13048-024-01544-8 39506832 PMC11539702

[B17] Chen J. Wang L. Tian G. G. Wang X. Li X. Wu J. (2023). Metformin promotes proliferation of mouse female germline stem cells by Histone Acetylation Modification of Traf2. Stem Cell Rev. Rep. 19 (7), 2329–2340. 10.1007/s12015-023-10575-5 37354386

[B18] Chen Y. H. Yang S. F. Yang C. K. Tsai H. D. Chen T. H. Chou M. C. (2021). Metformin induces apoptosis and inhibits migration by activating the AMPK/p53 axis and suppressing PI3K/AKT signaling in human cervical cancer cells. Mol. Med. Rep. 23 (1), 88. 10.3892/mmr.2020.11725 33236135 PMC7716426

[B19] Cheng D. Xu Q. Wang Y. Li G. Sun W. Ma D. (2021). Metformin attenuates silica-induced pulmonary fibrosis via AMPK signaling. J. Transl. Med. 19 (1), 349. 10.1186/s12967-021-03036-5 34399790 PMC8365894

[B20] Cheng J. Li C. Ying Y. Lv J. Qu X. McGowan E. (2022). Metformin alleviates endometriosis and potentiates endometrial Receptivity via decreasing VEGF and MMP9 and increasing leukemia inhibitor factor and HOXA10. Front. Pharmacol. 13, 750208. 10.3389/fphar.2022.750208 35273494 PMC8902464

[B21] Cheng K. Hao M. (2016). Metformin inhibits TGF-β1-induced epithelial-to-Mesenchymal Transition via PKM2 Relative-mTOR/p70s6k signaling pathway in cervical carcinoma cells. Int. J. Mol. Sci. 17 (12), 2000. 10.3390/ijms17122000 27916907 PMC5187800

[B22] Davis S. R. Robinson P. J. Jane F. White S. Brown K. A. Piessens S. (2018). The benefits of adding metformin to tamoxifen to protect the endometrium-A randomized placebo-controlled trial. Clin. Endocrinol. (Oxf) 89 (5), 605–612. 10.1111/cen.13830 30107043

[B23] Diamanti-Kandarakis E. Kouli C. Tsianateli T. Bergiele A. (1998). Therapeutic effects of metformin on insulin resistance and hyperandrogenism in polycystic ovary syndrome. Eur. J. Endocrinol. 138 (3), 269–274. 10.1530/eje.0.1380269 9539300

[B24] Di Tommaso S. Massari S. Malvasi A. Bozzetti M. P. Tinelli A. (2013). Gene expression analysis reveals an angiogenic profile in uterine leiomyoma pseudocapsule. Mol. Hum. Reprod. 19 (6), 380–387. 10.1093/molehr/gat007 23355533

[B25] Do M. T. Kim H. G. Khanal T. Choi J. H. Kim D. H. Jeong T. C. (2013). Metformin inhibits heme oxygenase-1 expression in cancer cells through inactivation of Raf-ERK-Nrf2 signaling and AMPK-independent pathways. Toxicol. Appl. Pharmacol. 271 (2), 229–238. 10.1016/j.taap.2013.05.010 23707609

[B26] Drevinskaite M. Kaceniene A. Linkeviciute-Ulinskiene D. Smailyte G. (2023). The impact of metformin on survival in diabetic endometrial cancer patients: a retrospective population-based analysis. J. Diabetes Metabolic Disord. 23, 841–847. 10.1007/s40200-023-01358-3 PMC11196484 38932795

[B27] Du D. Tang X. Li Y. Gao Y. Chen R. Chen Q. (2022). Senotherapy protects against cisplatin-induced ovarian injury by Removing senescent cells and alleviating DNA damage. Oxid. Med. Cell Longev. 2022, 9144644. 10.1155/2022/9144644 35693700 PMC9187433

[B28] Duleba A. J. Dokras A. (2012). Is PCOS an inflammatory process? Fertil. Steril. 97 (1), 7–12. 10.1016/j.fertnstert.2011.11.023 22192135 PMC3245829

[B29] Echiburu B. Perez-Bravo F. Galgani J. E. Sandoval D. Saldias C. Crisosto N. (2018). Enlarged adipocytes in subcutaneous adipose tissue associated to hyperandrogenism and visceral adipose tissue volume in women with polycystic ovary syndrome. Steroids 130, 15–21. 10.1016/j.steroids.2017.12.009 29273198

[B30] Ellibishy F. Tarek M. Abd-Elsalam M. M. Elgayar N. El Bakly W. (2024). Metformin improves d-galactose induced premature ovarian insufficiency through PI3K-Akt-FOXO3a pathway. Adv. Med. Sci. 69 (1), 70–80. 10.1016/j.advms.2024.02.004 38387407

[B31] Foretz M. Guigas B. Viollet B. (2023). Metformin: update on mechanisms of action and repurposing potential. Nat. Rev. Endocrinol. 19 (8), 460–476. 10.1038/s41574-023-00833-4 37130947 PMC10153049

[B32] Garcia-Beltran C. Malpique R. Andersen M. S. Bas F. Bassols J. Darendeliler F. (2023). SPIOMET4HEALTH-efficacy, tolerability and safety of lifestyle intervention plus a fixed dose combination of spironolactone, pioglitazone and metformin (SPIOMET) for adolescent girls and young women with polycystic ovary syndrome: study protocol for a multicentre, randomised, double-blind, placebo-controlled, four-arm, parallel-group, phase II clinical trial. Trials 24 (1), 589. 10.1186/s13063-023-07593-6 37715279 PMC10503102

[B33] George J. W. (2023). The burden of uterine fibroids: an overview. J. Histotechnol. 46 (4), 153–155. 10.1080/01478885.2023.2265185 37791571

[B34] Golezar S. Ramezani Tehrani F. Khazaei S. Ebadi A. Keshavarz Z. (2019). The global prevalence of primary ovarian insufficiency and early menopause: a meta-analysis. Climacteric 22 (4), 403–411. 10.1080/13697137.2019.1574738 30829083

[B35] Gonzalez F. Considine R. V. Abdelhadi O. A. Acton A. J. (2019). Oxidative stress in response to Saturated fat Ingestion is linked to insulin resistance and hyperandrogenism in polycystic ovary syndrome. J. Clin. Endocrinol. Metab. 104 (11), 5360–5371. 10.1210/jc.2019-00987 31298704 PMC6773460

[B36] Gonzalez F. Kirwan J. P. Rote N. S. Minium J. O'Leary V. B. (2014). Glucose and lipopolysaccharide regulate proatherogenic cytokine release from mononuclear cells in polycystic ovary syndrome. J. Reprod. Immunol. 103, 38–44. 10.1016/j.jri.2014.01.001 24576416 PMC4020957

[B37] Gu B. X. Wang X. Yin B. L. Guo H. B. Zhang H. L. Zhang S. D. (2016). Abnormal expression of TLRs may play a role in lower embryo quality of women with polycystic ovary syndrome. Syst. Biol. Reprod. Med. 62 (5), 353–358. 10.1080/19396368.2016.1187683 27367829

[B38] Guney G. Taskin M. I. Sener N. Tolu E. Dodurga Y. Elmas L. (2022). The role of ERK-1 and ERK-2 gene polymorphisms in PCOS pathogenesis. Reprod. Biol. Endocrinol. 20 (1), 95. 10.1186/s12958-022-00967-6 35768803 PMC9241270

[B39] Guo R. Zheng H. Li Q. Y. Qiu X. Zhang J. Cheng Z. F. (2022). Melatonin alleviates insulin resistance through the PI3K/AKT signaling pathway in ovary granulosa cells of polycystic ovary syndrome. Reprod. Biol. 22 (1), 100594. 10.1016/j.repbio.2021.100594 34953312

[B40] Han C. Y. Patten D. A. Lee S. G. Parks R. J. Chan D. W. Harper M. E. (2019). p53 Promotes chemoresponsiveness by regulating hexokinase II gene transcription and metabolic reprogramming in epithelial ovarian cancer. Mol. Carcinog. 58 (11), 2161–2174. 10.1002/mc.23106 31486135

[B41] Han K. Pintilie M. Lipscombe L. L. Lega I. C. Milosevic M. F. Fyles A. W. (2016). Association between metformin Use and mortality after cervical cancer in older women with diabetes. Cancer Epidemiol. Biomarkers and Prev. 25 (3), 507–512. 10.1158/1055-9965.EPI-15-1008 26721670

[B42] Hanna R. K. Zhou C. X. Malloy K. M. Sun L. Zhong Y. Gehrig P. A. (2012). Metformin potentiates the effects of paclitaxel in endometrial cancer cells through inhibition of cell proliferation and modulation of the mTOR pathway. Gynecol. Oncol. 125 (2), 458–469. 10.1016/j.ygyno.2012.01.009 22252099 PMC3322276

[B43] Hanprasertpong J. Jiamset I. Geater A. Peerawong T. Hemman W. Kornsilp S. (2017). The effect of metformin on Oncological outcomes in patients with cervical cancer with type 2 diabetes mellitus. Int. J. Gynecol. Cancer 27 (1), 131–137. 10.1097/IGC.0000000000000855 27870711

[B44] He Q. Wan S. Jiang M. Li W. Zhang Y. Zhang L. (2024). Exploring the therapeutic potential of tonic Chinese herbal medicine for gynecological disorders: an updated review. J. Ethnopharmacol. 329, 118144. 10.1016/j.jep.2024.118144 38583732

[B45] Hirsch A. Hahn D. Kempna P. Hofer G. Nuoffer J. M. Mullis P. E. (2012). Metformin inhibits human androgen production by regulating steroidogenic enzymes HSD3B2 and CYP17A1 and complex I activity of the respiratory chain. Endocrinology 153 (9), 4354–4366. 10.1210/en.2012-1145 22778212

[B46] Hong W. Wang B. Zhu Y. Wu J. Qiu L. Ling S. (2022). Female germline stem cells: aging and anti-aging. J. Ovarian Res. 15 (1), 79. 10.1186/s13048-022-01011-2 35787298 PMC9251950

[B47] Hu M. Zhang Y. Li X. Cui P. Sferruzzi-Perri A. N. Brannstrom M. (2021). TLR4-Associated IRF-7 and NFκB signaling Act as a molecular Link between androgen and metformin activities and cytokine synthesis in the PCOS endometrium. J. Clin. Endocrinol. Metab. 106 (4), 1022–1040. 10.1210/clinem/dgaa951 33382900

[B48] Hu M. Zhang Y. H. Feng J. X. Xu X. Zhang J. Zhao W. (2018). Uterine progesterone signaling is a target for metformin therapy in PCOS-like rats. J. Endocrinol. 237 (2), 123–137. 10.1530/JOE-18-0086 29535146

[B49] Huang C. C. Chou C. H. Yang Y. S. Ho H. N. Shun C. T. Wen W. F. (2021). Metformin: a novel promising option for fertility preservation during cyclophosphamide-based chemotherapy. Mol. Hum. Reprod. 27 (1), gaaa084. 10.1093/molehr/gaaa084 33543290 PMC8494485

[B50] Hyun B. Shin S. Lee A. Lee S. Song Y. Ha N. J. (2013). Metformin Down-regulates TNF-alpha secretion via suppression of Scavenger receptors in macrophages. Immune Netw. 13 (4), 123–132. 10.4110/in.2013.13.4.123 24009539 PMC3759709

[B51] Isoda K. Young J. L. Zirlik A. MacFarlane L. A. Tsuboi N. Gerdes N. (2006). Metformin inhibits proinflammatory responses and nuclear factor-kappaB in human vascular wall cells. Arterioscler. Thromb. Vasc. Biol. 26 (3), 611–617. 10.1161/01.ATV.0000201938.78044.75 16385087

[B52] Jamali N. Zal F. Mostafavi-Pour Z. Samare-Najaf M. Poordast T. Dehghanian A. (2021). Ameliorative effects of Quercetin and metformin and their combination against experimental endometriosis in rats. Reprod. Sci. 28 (3), 683–692. 10.1007/s43032-020-00377-2 33141412

[B53] Jia Y. Cui R. Wang C. Feng Y. Li Z. Tong Y. (2020). Metformin protects against intestinal ischemia-reperfusion injury and cell pyroptosis via TXNIP-NLRP3-GSDMD pathway. Redox Biol. 32, 101534. 10.1016/j.redox.2020.101534 32330868 PMC7178548

[B54] Jiang Y. Chen X. Wei Y. Feng Y. Zheng W. Zhang Z. (2019). Metformin sensitizes endometrial cancer cells to progestin by targeting TET1 to downregulate glyoxalase I expression. Biomed. Pharmacother. 113, 108712. 10.1016/j.biopha.2019.108712 30849641

[B55] Jin L. Jin F. Guo S. Liu W. Wei B. Fan H. (2022). Metformin inhibits NLR family pyrin domain containing 3 (NLRP)-Relevant Neuroinflammation via an Adenosine-5'-Monophosphate-activated protein kinase (AMPK)-Dependent pathway to alleviate early brain injury after Subarachnoid Hemorrhage in mice. Front. Pharmacol. 13, 796616. 10.3389/fphar.2022.796616 35370693 PMC8969021

[B56] Kim H. M. Kang M. J. Song S. O. (2022). Metformin and cervical cancer risk in patients with Newly diagnosed type 2 diabetes: a population-based study in Korea. Endocrinol. Metab. Seoul. 37 (6), 929–937. 10.3803/EnM.2022.1613 36604960 PMC9816509

[B57] Kim Y. C. Guan K. L. (2015). mTOR: a pharmacologic target for autophagy regulation. J. Clin. Invest. 125 (1), 25–32. 10.1172/JCI73939 25654547 PMC4382265

[B58] Kitson S. J. Maskell Z. Sivalingam V. N. Allen J. L. Ali S. Burns S. (2019). PRE-Surgical metformin in uterine malignancy (PREMIUM): a multi-center, randomized double-blind, placebo-controlled phase III trial. Clin. Cancer Res. 25 (8), 2424–2432. 10.1158/1078-0432.CCR-18-3339 30563932 PMC6586555

[B59] Kobayashi H. Imanaka S. Yoshimoto C. Matsubara S. Shigetomi H. (2024). Molecular mechanism of autophagy and apoptosis in endometriosis: current understanding and future research directions. Reprod. Med. Biol. 23 (1), e12577. 10.1002/rmb2.12577 38645639 PMC11031673

[B60] Kulkarni A. S. Gubbi S. Barzilai N. (2020). Benefits of metformin in attenuating the Hallmarks of aging. Cell Metab. 32 (1), 15–30. 10.1016/j.cmet.2020.04.001 32333835 PMC7347426

[B61] Kumar S. Meuter A. Thapa P. Langstraat C. Giri S. Chien J. (2013). Metformin intake is associated with better survival in ovarian cancer A Case-Control Study. Cancer 119 (3), 555–562. 10.1002/cncr.27706 23208739 PMC3553259

[B62] Kurzthaler D. Hadziomerovic-Pekic D. Wildt L. Seeber B. E. (2014). Metformin induces a prompt decrease in LH-stimulated testosterone response in women with PCOS independent of its insulin-sensitizing effects. Reprod. Biol. Endocrinol. 12, 98. 10.1186/1477-7827-12-98 25304843 PMC4199060

[B63] la Marca A. Morgante G. Paglia T. Ciotta L. Cianci A. De Leo V. (1999). Effects of metformin on adrenal steroidogenesis in women with polycystic ovary syndrome. Fertil. Steril. 72 (6), 985–989. 10.1016/s0015-0282(99)00407-0 10593368

[B64] LaMoia T. E. Shulman G. I. (2021). Cellular and molecular mechanisms of metformin action. Endocr. Rev. 42 (1), 77–96. 10.1210/endrev/bnaa023 32897388 PMC7846086

[B65] Lan C. W. Chen M. J. Tai K. Y. Yu D. C. Yang Y. C. Jan P. S. (2015). Functional microarray analysis of differentially expressed genes in granulosa cells from women with polycystic ovary syndrome related to MAPK/ERK signaling. Sci. Rep. 5, 14994. 10.1038/srep14994 26459919 PMC4602237

[B66] Landry D. A. Yakubovich E. Cook D. P. Fasih S. Upham J. Vanderhyden B. C. (2022). Metformin prevents age-associated ovarian fibrosis by modulating the immune landscape in female mice. Sci. Adv. 8 (35), eabq1475. 10.1126/sciadv.abq1475 36054356 PMC10848964

[B67] Lee J. An S. Jung J. H. Kim K. Kim J. Y. An I. S. (2019). MUL1 E3 ligase regulates the antitumor effects of metformin in chemoresistant ovarian cancer cells via AKT degradation. Int. J. Oncol. 54 (5), 1833–1842. 10.3892/ijo.2019.4730 30816444

[B68] Lee T. Y. Martinez-Outschoorn U. E. Schilder R. J. Kim C. H. Richard S. D. Rosenblum N. G. (2018). Metformin as a therapeutic target in endometrial cancers. Front. Oncol. 8, 341. 10.3389/fonc.2018.00341 30211120 PMC6121131

[B69] Li B. Takeda T. Tsuiji K. Kondo A. Kitamura M. Wong T. F. (2013). The antidiabetic drug metformin inhibits uterine leiomyoma cell proliferation via an AMP-activated protein kinase signaling pathway. Gynecol. Endocrinol. 29 (1), 87–90. 10.3109/09513590.2012.706668 22835064

[B70] Li C. Zhao H. L. Li Y. J. Zhang Y. Y. Liu H. Y. Feng F. Z. (2021). The expression and significance of leukemia inhibitory factor, interleukin-6 and vascular endothelial growth factor in Chinese patients with endometriosis. Arch. Gynecol. Obstet. 304 (1), 163–170. 10.1007/s00404-021-05980-5 33555431

[B71] Li H. Yang H. D. Lu S. Y. Wang X. Y. Shi X. H. Mao P. Y. (2023). Autophagy-dependent ferroptosis is involved in the development of endometriosis. Gynecol. Endocrinol. 39 (1), 2242962. 10.1080/09513590.2023.2242962 37553011

[B72] Li J. Qin Z. Li Y. Huang B. Xiao Q. Chen P. (2024). Phosphorylation of IDH1 facilitates progestin resistance in endometrial cancer. Adv. Sci. (Weinh) 11, e2310208. 10.1002/advs.202310208 38582508 PMC11187910

[B75] Li Y. Liu X. Wan L. Han B. Ma S. Pan H. (2023). Metformin suppresses cardiac fibroblast proliferation under high-glucose conditions via regulating the mitochondrial complex I protein Grim-19 involved in the Sirt1/Stat3 signaling pathway. Free Radic. Biol. Med. 206, 1–12. 10.1016/j.freeradbiomed.2023.06.013 37353174

[B76] Li Y. Pei T. Zhu H. Wang R. Wu L. Huang X. (2025). Melatonin alleviates Circadian Rhythm disruption-induced enhanced luteinizing hormone Pulse frequency and ovarian dysfunction. J. Pineal Res. 77 (1), e70026. 10.1111/jpi.70026 39757996

[B77] Lin L. Gao W. Chen Y. Li T. Sha C. Chen L. (2022). Reactive oxygen species-induced SIAH1 promotes granulosa cells' senescence in premature ovarian failure. J. Cell Mol. Med. 26 (8), 2417–2427. 10.1111/jcmm.17264 35261172 PMC8995443

[B78] Liu H. Zhang Z. Xiong W. Zhang L. Xiong Y. Li N. (2017). Hypoxia-inducible factor-1α promotes endometrial stromal cells migration and invasion by upregulating autophagy in endometriosis. Reproduction 153 (6), 809–820. 10.1530/REP-16-0643 28348069 PMC5489654

[B79] Liu N. T. Perng C. L. Chou Y. C. Ko P. S. Lin Y. J. Lin Y. C. (2021). Loss of ten-eleven translocation 1 (TET1) expression as a diagnostic and prognostic biomarker of endometrial carcinoma. Plos One 16 (11), e0259330. 10.1371/journal.pone.0259330 34731191 PMC8565757

[B80] Liu Y. Feng Y. Liu H. Wu J. Tang Y. Wang Q. (2018). Real-time assessment of platinum sensitivity of primary culture from a patient with ovarian cancer with extensive metastasis and the platinum sensitivity enhancing effect by metformin. Oncol. Lett. 16 (4), 4253–4262. 10.3892/ol.2018.9223 30250536 PMC6144930

[B81] Liu Y. Wang J. Zhang X. (2022). An update on the multifaceted role of NF-kappaB in endometriosis. Int. J. Biol. Sci. 18 (11), 4400–4413. 10.7150/ijbs.72707 35864971 PMC9295070

[B82] Lonardo M. S. Cacciapuoti N. Guida B. Di Lorenzo M. Chiurazzi M. Damiano S. (2024). Hypothalamic-ovarian axis and adiposity relationship in polycystic ovary syndrome: Physiopathology and therapeutic options for the management of metabolic and inflammatory Aspects. Curr. Obes. Rep. 13 (1), 51–70. 10.1007/s13679-023-00531-2 38172476 PMC10933167

[B83] Lousse J. C. Van Langendonckt A. Gonzalez-Ramos R. Defrere S. Renkin E. Donnez J. (2008). Increased activation of nuclear factor-kappa B (NF-kappaB) in isolated peritoneal macrophages of patients with endometriosis. Fertil. Steril. 90 (1), 217–220. 10.1016/j.fertnstert.2007.06.015 17889859

[B84] Lu G. Wu Z. Shang J. Xie Z. Chen C. Zhang C. (2021). The effects of metformin on autophagy. Biomed. Pharmacother. 137, 111286. 10.1016/j.biopha.2021.111286 33524789

[B85] Lu X. E. Ning W. X. Dong M. Y. Liu A. X. Jin F. Huang H. F. (2006). Vascular endothelial growth factor and matrix metalloproteinase-2 expedite formation of endometriosis in the early stage ICR mouse model. Fertil. Steril. 86 (4 Suppl. l), 1175–1181. 10.1016/j.fertnstert.2005.12.083 16962110

[B86] Lv Q. Y. Xie B. Y. Yang B. Y. Ning C. C. Shan W. W. Gu C. (2017). Increased TET1 expression in inflammatory Microenvironment of Hyperinsulinemia enhances the response of endometrial cancer to estrogen by epigenetic modulation of GPER. J. Cancer 8 (5), 894–902. 10.7150/jca.17064 28382153 PMC5381179

[B87] Maoga J. B. Riaz M. A. Mwaura A. N. Mecha E. Omwandho C. O. A. Scheiner-Bobis G. (2023). Analysis of Membrane type-1 matrix metalloproteinase (MT1-MMP, MMP14) in Eutopic and ectopic endometrium and in serum and Endocervical Mucus of endometriosis. Biomedicines 11 (10), 2730. 10.3390/biomedicines11102730 37893104 PMC10604514

[B88] Martins A. F. Neto A. C. Rodrigues A. R. Oliveira S. M. Sousa-Mendes C. Leite-Moreira A. (2022). Metformin prevents endothelial dysfunction in endometriosis through downregulation of ET-1 and upregulation of eNOS. Biomedicines 10 (11), 2782. 10.3390/biomedicines10112782 36359302 PMC9687337

[B89] McCloskey C. W. Cook D. P. Kelly B. S. Azzi F. Allen C. H. Forsyth A. (2020). Metformin Abrogates age-associated ovarian fibrosis. Clin. Cancer Res. 26 (3), 632–642. 10.1158/1078-0432.CCR-19-0603 31597663

[B90] Meirow D. Dor J. Kaufman B. Shrim A. Rabinovici J. Schiff E. (2007). Cortical fibrosis and blood-vessels damage in human ovaries exposed to chemotherapy. Potential mechanisms of ovarian injury. Hum. Reprod. 22 (6), 1626–1633. 10.1093/humrep/dem027 17324957

[B91] Min X. Zhang T. Lin Y. Wang B. Zhu K. (2020). Metformin inhibits the growth of ovarian cancer cells by promoting the Parkin-induced p53 ubiquitination. Biosci. Rep. 10.1042/BSR20200679 32869837

[B92] Moghetti P. Castello R. Negri C. Tosi F. Perrone F. Caputo M. (2000). Metformin effects on clinical features, endocrine and metabolic profiles, and insulin sensitivity in polycystic ovary syndrome: a randomized, double-blind, placebo-controlled 6-month trial, followed by open, long-term clinical evaluation. J. Clin. Endocrinol. Metab. 85 (1), 139–146. 10.1210/jcem.85.1.6293 10634377

[B93] Mohebbi A. Hojati V. Majidi Zolbin M. Aflatoonian R. (2022). Histopathologic evaluation of the inflammatory factors and stromal cells in the endometriosis lesions: a case-control study. Int. J. Reprod. Biomed. 20 (10), 819–830. 10.18502/ijrm.v20i10.12266 36381357 PMC9644650

[B94] Moravek M. B. Bulun S. E. (2015). Endocrinology of uterine fibroids: steroid hormones, stem cells, and genetic contribution. Curr. Opin. Obstet. Gynecol. 27 (4), 276–283. 10.1097/GCO.0000000000000185 26107781 PMC4734398

[B95] Mousa M. Al-Jefout M. Alsafar H. Kirtley S. Lindgren C. M. Missmer S. A. (2021). Prevalence of common gynecological conditions in the Middle East: systematic review and meta-analysis. Front. Reprod. Health 3, 661360. 10.3389/frph.2021.661360 36304010 PMC9580651

[B96] Ojavee S. E. Darrous L. Patxot M. Läll K. Fischer K. Mägi R. (2023). Genetic insights into the age-specific biological mechanisms governing human ovarian aging. Am. J. Hum. Genet. 110 (9), 1549–1563. 10.1016/j.ajhg.2023.07.006 37543033 PMC10502738

[B97] Omer N. A. T. M. A. Aljebory H. D. (2016). Effect of metformin treatment on some blood Biomarkers in women with endometriosis. Iraqi J. Pharm. Sci. 25 (1), 28–36. 10.31351/vol25iss1pp28-36

[B98] Peitsidis P. Tsikouras P. Lagana A. S. Laios A. Gkegkes I. D. Iavazzo C. (2023). A systematic review of systematic reviews on the Use of aromatase inhibitors for the treatment of endometriosis: the evidence to date. Drug Des. Devel Ther. 17, 1329–1346. 10.2147/DDDT.S315726 PMC10166210 37168488

[B99] Pellicer N. Cozzolino M. Diaz-Garcia C. Galliano D. Cobo A. Pellicer A. (2023). Ovarian rescue in women with premature ovarian insufficiency: facts and fiction. Reprod. Biomed. Online 46 (3), 543–565. 10.1016/j.rbmo.2022.12.011 36710157

[B100] Persani L. Rossetti R. Cacciatore C. Fabre S. (2011). Genetic defects of ovarian TGF-beta-like factors and premature ovarian failure. J. Endocrinol. Invest. 34 (3), 244–251. 10.1007/BF03347073 21297384

[B101] Petrasca A. Hambly R. Kearney N. Smith C. M. Pender E. K. Mac Mahon J. (2023). Metformin has anti-inflammatory effects and induces immunometabolic reprogramming via multiple mechanisms in hidradenitis suppurativa. Br. J. Dermatol 189 (6), 730–740. 10.1093/bjd/ljad305 37648653 PMC13077222

[B102] Pinyopornpanish K. Leerapun A. Pinyopornpanish K. Chattipakorn N. (2021). Effects of metformin on hepatic Steatosis in Adults with Nonalcoholic fatty liver disease and diabetes: insights from the cellular to patient levels. Gut Liver 15 (6), 827–840. 10.5009/gnl20367 33820884 PMC8593497

[B103] Proteau-Lemieux M. Lacroix A. Galarneau L. Corbin F. Lepage J. F. Caku A. (2021). The safety and efficacy of metformin in fragile X syndrome: an open-label study. Prog. Neuropsychopharmacol. Biol. Psychiatry 110, 110307. 10.1016/j.pnpbp.2021.110307 33757860

[B104] Qin X. Du D. Chen Q. Wu M. Wu T. Wen J. (2019). Metformin prevents murine ovarian aging. Aging (Albany NY) 11 (11), 3785–3794. 10.18632/aging.102016 31182682 PMC6594816

[B105] Rabah H. M. Mohamed D. A. Mariah R. A. Abd El-Khalik S. R. Khattab H. A. AbuoHashish N. A. (2023). Novel insights into the synergistic effects of selenium nanoparticles and metformin treatment of letrozole - induced polycystic ovarian syndrome: targeting PI3K/Akt signalling pathway, redox status and mitochondrial dysfunction in ovarian tissue. Redox Rep. 28 (1), 2160569. 10.1080/13510002.2022.2160569 36661246 PMC9870018

[B106] Rattan R. Graham R. P. Maguire J. L. Giri S. Shridhar V. (2011). Metformin suppresses ovarian cancer growth and metastasis with enhancement of cisplatin cytotoxicity *in vivo* . Neoplasia 13 (5), 483–491. 10.1593/neo.11148 21532889 PMC3084625

[B107] Ricci F. Brunelli L. Affatato R. Chila R. Verza M. Indraccolo S. (2019). Overcoming platinum-acquired resistance in ovarian cancer patient-derived xenografts. Ther. Adv. Med. Oncol. 11, 1758835919839543. 10.1177/1758835919839543 31258626 PMC6591669

[B108] Rice S. Elia A. Jawad Z. Pellatt L. Mason H. D. (2013). Metformin inhibits follicle-stimulating hormone (FSH) action in human granulosa cells: relevance to polycystic ovary syndrome. J. Clin. Endocrinol. Metab. 98 (9), E1491–E1500. 10.1210/jc.2013-1865 23846817 PMC3784648

[B109] Rudnicka E. Duszewska A. M. Kucharski M. Tyczynski P. Smolarczyk R. (2022). Oxidative stress and reproductive function: oxidative stress in polycystic ovary syndrome. Reproduction 164 (6), F145–F154. 10.1530/REP-22-0152 36279177

[B110] Ruiz-Mitjana A. Vidal-Sabanes M. Navaridas R. Perramon-Guell A. Yeramian A. Nicholson-Sabate N. (2023). Metformin exhibits antineoplastic effects on Pten-deficient endometrial cancer by interfering with TGF-beta and p38/ERK MAPK signalling. Biomed. Pharmacother. 168, 115817. 10.1016/j.biopha.2023.115817 37925934

[B112] Saber S. El-Kader E. M. A. (2021). Novel complementary coloprotective effects of metformin and MCC950 by modulating HSP90/NLRP3 interaction and inducing autophagy in rats. Inflammopharmacology 29 (1), 237–251. 10.1007/s10787-020-00730-6 32594364

[B113] Sapmaz T. Coskun G. Saker D. Pence H. H. Keles P. Hayretdag C. (2022). Effects of metformin, letrozole and atorvastatin on inflammation and apoptosis in experimental peritoneal and ovarian endometriosis in the rat. Pathol. Res. Pract. 235, 153951. 10.1016/j.prp.2022.153951 35644046

[B114] Sarfstein R. Friedman Y. Attias-Geva Z. Fishman A. Bruchim I. Werner H. (2013). Metformin downregulates the insulin/IGF-I signaling pathway and inhibits different uterine serous carcinoma (USC) cells proliferation and migration in p53-dependent or -independent manners. PLoS One 8 (4), e61537. 10.1371/journal.pone.0061537 23620761 PMC3631250

[B115] Shi J. Liu B. Wang H. Zhang T. Yang L. (2019). Association of metformin use with ovarian cancer incidence and prognosis: a systematic review and meta-analysis. Int. J. Gynecol. Cancer 29 (1), 140–146. 10.1136/ijgc-2018-000060 30640696

[B116] Skov V. Glintborg D. Knudsen S. Jensen T. Kruse T. A. Tan Q. (2007). Reduced expression of nuclear-encoded genes involved in mitochondrial oxidative metabolism in skeletal muscle of insulin-resistant women with polycystic ovary syndrome. Diabetes 56 (9), 2349–2355. 10.2337/db07-0275 17563058

[B117] Sumitani H. Shozu M. Segawa T. Murakami K. Yang H. J. Shimada K. (2000). *In situ* estrogen synthesized by aromatase P450 in uterine leiomyoma cells promotes cell growth probably via an autocrine/intracrine mechanism. Endocrinology 141 (10), 3852–3861. 10.1210/endo.141.10.7719 11014242

[B118] Syngelaki A. Nicolaides K. H. Balani J. Hyer S. Akolekar R. Kotecha R. (2016). Metformin versus placebo in obese pregnant women without diabetes mellitus. N. Engl. J. Med. 374 (5), 434–443. 10.1056/NEJMoa1509819 26840133

[B119] Tadakawa M. Takeda T. Li B. Tsuiji K. Yaegashi N. (2015). The anti-diabetic drug metformin inhibits vascular endothelial growth factor expression via the mammalian target of rapamycin complex 1/hypoxia-inducible factor-1α signaling pathway in ELT-3 cells. Mol. Cell Endocrinol. 399, 1–8. 10.1016/j.mce.2014.08.012 25179820

[B120] Takemura Y. Osuga Y. Yoshino O. Hasegawa A. Hirata T. Hirota Y. (2007). Metformin suppresses interleukin (IL)-1beta-induced IL-8 production, aromatase activation, and proliferation of endometriotic stromal cells. J. Clin. Endocrinol. Metab. 92 (8), 3213–3218. 10.1210/jc.2006-2486 17504902

[B121] Takiuchi T. Machida H. Hom M. S. Mostofizadeh S. Frimer M. Brunette L. L. (2017). Association of metformin Use and survival outcome in women with cervical cancer. Int. J. Gynecol. Cancer 27 (7), 1455–1463. 10.1097/IGC.0000000000001036 29049093 PMC7526033

[B122] Tang Y. L. Zhu L. Y. Li Y. Yu J. Wang J. Zeng X. X. (2017). Metformin Use is associated with reduced incidence and improved survival of endometrial cancer: a meta-analysis. Biomed Res. Int. 2017, 5905384. 10.1155/2017/5905384 28409158 PMC5376924

[B123] Taylor H. S. Kotlyar A. M. Flores V. A. (2021). Endometriosis is a chronic systemic disease: clinical challenges and novel innovations. Lancet 397 (10276), 839–852. 10.1016/S0140-6736(21)00389-5 33640070

[B124] Tong S. Kaitu'u-Lino T. J. Hastie R. Brownfoot F. Cluver C. Hannan N. (2022). Pravastatin, proton-pump inhibitors, metformin, micronutrients, and biologics: new horizons for the prevention or treatment of preeclampsia. Am. J. Obstet. Gynecol. 226 (2S), S1157–S1170. 10.1016/j.ajog.2020.09.014 32946849

[B125] Top W. M. C. Kooy A. Stehouwer C. D. A. (2022). Metformin: a Narrative review of its potential benefits for cardiovascular disease, cancer and Dementia. Pharm. (Basel) 15 (3), 312. 10.3390/ph15030312 PMC8951049 35337110

[B126] Tseng C. H. (2015). Metformin reduces ovarian cancer risk in Taiwanese women with type 2 diabetes mellitus. Diabetes Metab. Res. Rev. 31 (6), 619–626. 10.1002/dmrr.2649 25820555

[B127] Tseng C. H. (2019). Metformin use is associated with a lower risk of uterine leiomyoma in female type 2 diabetes patients. Ther. Adv. Endocrinol. Metabolism 10, 2042018819895159. 10.1177/2042018819895159 PMC6920594 31897287

[B128] Tyszka-Czochara M. Bukowska-Strakova K. Majka M. (2017). Metformin and caffeic acid regulate metabolic reprogramming in human cervical carcinoma SiHa/HTB-35 cells and augment anticancer activity of Cisplatin via cell cycle regulation. Food Chem. Toxicol. 106 (Pt A), 260–272. 10.1016/j.fct.2017.05.065 28576465

[B129] Udono H. Nishida M. (2022). Metformin-ROS-Nrf2 connection in the host defense mechanism against oxidative stress, apoptosis, cancers, and ageing. Biochim. Biophys. Acta Gen. Subj. 1866 (8), 130171. 10.1016/j.bbagen.2022.130171 35588955

[B130] Umehara T. Winstanley Y. E. Andreas E. Morimoto A. Williams E. J. Smith K. M. (2022). Female reproductive life span is extended by targeted removal of fibrotic collagen from the mouse ovary. Sci. Adv. 8 (24), eabn4564. 10.1126/sciadv.abn4564 35714185 PMC9205599

[B131] Vannuccini S. Clemenza S. Rossi M. Petraglia F. (2022). Hormonal treatments for endometriosis: the endocrine background. Rev. Endocr. Metab. Disord. 23 (3), 333–355. 10.1007/s11154-021-09666-w 34405378 PMC9156507

[B132] Velazquez C. Herrero Y. Bianchi M. S. Cohen D. J. Cuasnicu P. Prost K. (2023). Beneficial effects of metformin on mice female fertility after a high-fat diet intake. Mol. Cell Endocrinol. 575, 111995. 10.1016/j.mce.2023.111995 37364632

[B133] Victor V. M. Rovira-Llopis S. Banuls C. Diaz-Morales N. Lopez-Domenech S. Escribano-Lopez I. (2015). Metformin modulates human leukocyte/endothelial cell interactions and proinflammatory cytokines in polycystic ovary syndrome patients. Atherosclerosis 242 (1), 167–173. 10.1016/j.atherosclerosis.2015.07.017 26188541

[B134] Vrbikova J. Hill M. Starka L. Cibula D. Bendlova B. Vondra K. (2001). The effects of long-term metformin treatment on adrenal and ovarian steroidogenesis in women with polycystic ovary syndrome. Eur. J. Endocrinol. 144 (6), 619–628. 10.1530/eje.0.1440619 11375796

[B135] Wang D. Zhang Y. Cui L. Yang Q. Wang J. (2022). Elevated latent transforming growth factor beta binding protein 2 in endometriosis promotes endometrial stromal cell invasion and proliferation via the NF-kB signaling pathway. Mol. Cell Endocrinol. 550, 111647. 10.1016/j.mce.2022.111647 35429597

[B136] Wang F. Yan Y. C. Wang D. Y. Fan Q. N. Yi F. Y. Yang X. Y. (2024). Effects of Metformin on CIMT and FMD in PCOS patients: a systematic review and meta-analysis. Bmc Womens Health 24 (1), 426. 10.1186/s12905-024-03275-w 39061005 PMC11282760

[B138] Wang S. Y. Xue Q. Zhou Y. F. Yin L. (2019). Effects of metformin on the expression of estrogen synthetase and ER mRNA in uterine leiomyoma tissues. Zhonghua Fu Chan Ke Za Zhi 54 (4), 249–254. 10.3760/cma.j.issn.0529-567x.2019.04.007 31006191

[B139] Wang X. Wang H. Yi P. Baker C. Casey G. Xie X. (2023). Metformin restrains ZIKV replication and alleviates virus-induced inflammatory responses in microglia. Int. Immunopharmacol. 121, 110512. 10.1016/j.intimp.2023.110512 37343373

[B140] Wu M. Y. Chao K. H. Yang J. H. Lee T. H. Yang Y. S. Ho H. N. (2003). Nitric oxide synthesis is increased in the endometrial tissue of women with endometriosis. Hum. Reprod. 18 (12), 2668–2671. 10.1093/humrep/deg484 14645189

[B141] Wu Y. Huang J. Chen H. Tao H. He Y. Yang G. (2023). Tumor-derived oxidative stress triggers ovarian follicle loss in Breast cancer. Am. J. Pathol. 193 (5), 608–623. 10.1016/j.ajpath.2023.01.015 36804378

[B142] Xia C. Liang S. He Z. Zhu X. Chen R. Chen J. (2018). Metformin, a first-line drug for type 2 diabetes mellitus, disrupts the MALAT1/miR-142-3p sponge to decrease invasion and migration in cervical cancer cells. Eur. J. Pharmacol. 830, 59–67. 10.1016/j.ejphar.2018.04.027 29704494

[B143] Xia C. Liu C. He Z. Cai Y. Chen J. (2020). Metformin inhibits cervical cancer cell proliferation by modulating PI3K/Akt-induced major histocompatibility complex class I-related chain A gene expression. J. Exp. Clin. Cancer Res. 39 (1), 127. 10.1186/s13046-020-01627-6 32631421 PMC7336474

[B144] Xie H. Li M. Zheng Y. (2024). Associations of metformin therapy treatment with endometrial cancer risk and prognosis: a systematic review and meta-analysis. Gynecol. Oncol. 182, 15–23. 10.1016/j.ygyno.2024.01.007 38246042

[B145] Xing C. Lv B. Zhao H. Wang D. Li X. He B. (2021). Metformin and exenatide upregulate hepatocyte nuclear factor-4α, sex hormone binding globulin levels and improve hepatic triglyceride deposition in polycystic ovary syndrome with insulin resistance rats. J. Steroid Biochem. Mol. Biol. 214, 105992. 10.1016/j.jsbmb.2021.105992 34478829

[B146] Xu B. Dai W. Liu L. Han H. Zhang J. Du X. (2022). Metformin ameliorates polycystic ovary syndrome in a rat model by decreasing excessive autophagy in ovarian granulosa cells via the PI3K/AKT/mTOR pathway. Endocr. J. 69 (7), 863–875. 10.1507/endocrj.EJ21-0480 35228471

[B147] Xu J. N. Zeng C. Zhou Y. Peng C. Zhou Y. F. Xue Q. (2014). Metformin inhibits StAR expression in human endometriotic stromal cells via AMPK-mediated disruption of CREB-CRTC2 complex formation. J. Clin. Endocrinol. Metab. 99 (8), 2795–2803. 10.1210/jc.2014-1593 24823468

[B148] Yang C. Zhao N. Li D. Zou G. Chen Y. (2019). Metformin improves the sensitivity of ovarian cancer cells to chemotherapeutic agents. Oncol. Lett. 18 (3), 2404–2411. 10.3892/ol.2019.10564 31402943 PMC6676676

[B149] Yang X. Huang M. Zhang Q. Chen J. Li J. Han Q. (2021). Metformin Antagonizes ovarian cancer cells malignancy through MSLN mediated IL-6/STAT3 signaling. Cell Transplant. 30, 9636897211027819. 10.1177/09636897211027819 34238029 PMC8274104

[B150] Yang Y. Tang X. Yao T. Zhang Y. Zhong Y. Wu S. (2024). Metformin protects ovarian granulosa cells in chemotherapy-induced premature ovarian failure mice through AMPK/PPAR-γ/SIRT1 pathway. Sci. Rep. 14 (1), 1447. 10.1038/s41598-024-51990-z 38228655 PMC10791659

[B151] Yari S. Khoei H. H. Saber M. Esfandiari F. Moini A. Shahhoseini M. (2021). Metformin attenuates expression of angiogenic and inflammatory genes in human endometriotic stromal cells. Exp. Cell Res. 404 (2), 112659. 10.1016/j.yexcr.2021.112659 34022204

[B152] Yasmeen A. Beauchamp M. C. Piura E. Segal E. Pollak M. Gotlieb W. H. (2011). Induction of apoptosis by metformin in epithelial ovarian cancer: involvement of the Bcl-2 family proteins. Gynecol. Oncol. 121 (3), 492–498. 10.1016/j.ygyno.2011.02.021 21388661

[B153] Yilmaz B. Sucak A. Kilic S. Aksakal O. Aksoy Y. Lortlar N. (2010). Metformin regresses endometriotic implants in rats by improving implant levels of superoxide dismutase, vascular endothelial growth factor, tissue inhibitor of metalloproteinase-2, and matrix metalloproteinase-9. Am. J. Obstet. Gynecol. 202 (4), 368 e361–e8. 10.1016/j.ajog.2009.10.873 20035912

[B154] Yu H. Sun J. L. Hu H. L. (2024). Prophylactic administration of metformin reduces gestational diabetes mellitus incidence in the high-risk populations: a meta-analysis Metformin for gestational diabetes prevention. Ir. J. Med. Sci. 193 (1), 199–209. 10.1007/s11845-023-03380-z 37248332

[B155] Zafrakas M. Kotronis K. Papasozomenou P. Eskitzis P. Grimbizis G. (2020). Extracellular matrix metalloproteinases in the etiopathogenesis of endometriosis: a systematic review and critical appraisal. Clin. Exp. Obstetrics and Gynecol. 47 (2), 147–153. 10.31083/j.ceog.2020.02.5140

[B156] Zhang L. Liu H. Xiong W. He H. Fu T. Long X. (2024). CircFOXO3 mediates hypoxia-induced autophagy of endometrial stromal cells in endometriosis. FASEB J. 38 (5), e23515. 10.1096/fj.202301654RR 38470367

[B157] Zhang Y. Hu M. Meng F. Sun X. Xu H. Zhang J. (2017). Metformin ameliorates uterine defects in a rat model of polycystic ovary syndrome. EBioMedicine 18, 157–170. 10.1016/j.ebiom.2017.03.023 28336389 PMC5405166

[B158] Zhao H. Zhang J. Cheng X. Nie X. He B. (2023). Insulin resistance in polycystic ovary syndrome across various tissues: an updated review of pathogenesis, evaluation, and treatment. J. Ovarian Res. 16 (1), 9. 10.1186/s13048-022-01091-0 36631836 PMC9832677

[B159] Zheng Y. Zhang H. Sun H. (2022). Metformin inhibits the proliferation and invasion of ovarian cancer cells by suppressing tripartite motif-containing 37-induced tumor necrosis factor receptor-associated factor 2 ubiquitination. Cancer Sci. 113 (11), 3776–3786. 10.1111/cas.15524 35950370 PMC9633302

[B160] Zhou W. J. Yang H. L. Shao J. Mei J. Chang K. K. Zhu R. (2019). Anti-inflammatory cytokines in endometriosis. Cell Mol. Life Sci. 76 (11), 2111–2132. 10.1007/s00018-019-03056-x 30826860 PMC11105498

[B161] Zhou Y. Xu J. N. Zeng C. Li X. Zhou Y. F. Qi Y. (2015). Metformin suppresses prostaglandin E2-induced cytochrome P450 aromatase gene expression and activity via stimulation of AMP-activated protein kinase in human endometriotic stromal cells. Reprod. Sci. 22 (9), 1162–1170. 10.1177/1933719115590664 26058395

[B162] Zhu D. Chen Y. Huang J. Deng H. Shen X. Lu D. (2022). Effects of metformin on pregnancy outcome, metabolic profile, and sex hormone levels in women with polycystic ovary syndrome and their offspring: a systematic review and meta-analysis. Ann. Transl. Med. 10 (7), 418. 10.21037/atm-22-909 35530948 PMC9073771

[B163] Zhuo Z. Wang A. Yu H. (2016). Metformin targeting autophagy overcomes progesterone resistance in endometrial carcinoma. Arch. Gynecol. Obstet. 294 (5), 1055–1061. 10.1007/s00404-016-4148-0 27402506

